# A male congenital pelvic arteriovenous malformation diagnosed by abdominal ultrasound: A case report and literature review

**DOI:** 10.3389/fsurg.2022.907234

**Published:** 2023-01-09

**Authors:** Yanhua Huang, Xiatian Liu, Hongwei Qian

**Affiliations:** ^1^ Department of Ultrasound, Shaoxing People's Hospital, Shaoxing, China; ^2^ Department of Hepatobiliary and Pancreatic Surgery, Shaoxing People's Hospital, Shaoxing, China; ^3^ Shaoxing Key Laboratory of Minimally Invasive Abdominal Surgery and Precise Treatment of Tumor, Shaoxing, China

**Keywords:** congenital arteriovenous malformation, male, pelvis, urology, case report, ultrasound

## Abstract

Congenital pelvic arteriovenous malformation (AVM) is a rare vascular abnormality whereby arteries and veins are directly connected with malformed vascular plexus. Owing to its low incidence and nonspecific symptoms, the ultrasonographic characteristics of congenital pelvic AVM in males have been infrequently studied. A 30-year-old man visited our hospital complaining of progressive pain in the right lower abdomen and lumbar area since 2 months previously. Abdominal ultrasound (US) was performed at the initial examination and pelvic AVM was diagnosed, which was then confirmed by computed tomographic angiography. After right internal iliac artery embolization, the patient recovered uneventfully and remained asymptomatic during the 12-month follow-up period. Congenital pelvic AVM should thus be included in the differential diagnosis of pelvic cystic masses in males despite its low incidence, with US also being of great diagnostic value. We describe the ultrasonic features of AVM in detail and hope that this study may contribute to the ultrasonic diagnosis of congenital pelvic AVM in males.

## Introduction

Pelvic arteriovenous malformation (AVM) is a rare vascular abnormality whereby arteries and veins are connected with a nidus of dysplastic vascular channels, and it is particularly rare in male patients (1). As most pelvic AVMs are secondary to trauma, prior surgery, or tumor, congenital pelvic AVMs in males are even rarer (2–4).

There have been some studies on congenital pelvic AVM in the last few decades despite its low incidence. However, previous studies (Table 1) mainly affirmed the value of computed tomography (CT) and angiography, whereas ultrasound (US) was rarely characterized in detail despite it being the preferred initial examination owing to the advantages of low cost, convenience and easy operation. Moreover, the symptoms of pelvic AVM are nonspecific and include flank, abdominal or pelvic pain, hematuria, hemospermia, impotence, and dysuria (5), leading to difficulties in diagnosis. Therefore, the ultrasonographic characteristics of congenital pelvic AVM in male patients have been infrequently studied by clinicians.

**Table 1:** Synopsis of the presenting symptoms, diagnostic modalities, and therapeutic strategies of the eight congenital pelvic arteriovenous malformations developed in male patients.

Authors	Year	Age	Symptoms	Diagnostic modality	Treatment
Suzuki et al. (6)	2007	60	Hematospermia	CT	Steel coil embolization
Richards et al. (7)	2008	63	Moderately bothersome obstructive and irritative lower urinary tract symptoms	Magnetic resonance angiogram	Angioembolization
Hammad et al. (8)	2010	39	Inability to void urine	CT	Embolization
Mangold et al. (9)	2011	40	A hyperthermic swelling in the left buttock and upper thigh	CT	Coil embolization
Suzuki et al. (5)	2012	69	Voiding difficulty	Transrectal ultrasonography	Conservative treatment
Erbahceci Salik et al. (10)	2014	46	Progressively increasing pelvic pain for 1 year and exacerbation for 1 month	CTA	Embolization with Squid-12 and metallic coils
Addo et al. (11)	2015	21	Generalized abdominal pain and gross hematuria for 5 days	CT	Embolization with ethylene vinyl alcohol copolymer
Zabicki et al. (12)	2019	24	Intense pelvic pain radiating to the perineal area, occurring as a result of sexual intercourse and ejaculation	CTA	Embolization with pushable coils and thrombin

CT, computed tomography; CTA, computed tomographic angiography.

Against this background, we present a rare case of congenital pelvic AVM diagnosed by abdominal US and discuss the ultrasonic features for diagnosis. This work may contribute to the ultrasonic diagnosis of congenital pelvic AVM in the male population.

## Case presentation

**Figure 1:**
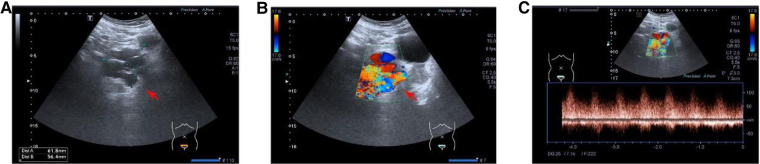
(**A**) Multiple tortuous tubular structures within an irregularly shaped anechoic lesion were revealed by B-mode ultrasound. (**B**) Color Doppler flow imaging displayed rich colorful blood flow signals as a colored mosaic pattern. (**C**) Spectral Doppler showed continuous turbulent flow in the systolic and diastolic phases with a high-velocity low-resistance waveform.

A 30-year-old man visited our hospital with a complaint of progressive persistent colic in the right lower abdomen and lumbar area over a period of 2 months, with no fever, nausea, vomiting, frequency/urgency of urination, urodynia, or hematuria. The causative factor was unclear. The patient denied a history of tumor, trauma, or previous surgery. Physical examination was normal with no tenderness or rebound tenderness in the abdomen, and no palpable mass was found. Laboratory investigations (routine blood and biochemical analysis, and tumor markers) were unremarkable. As the initial imaging examination, abdominal US revealed multiple tortuous tubular structures within an irregularly shaped anechoic lesion (62 mm × 56 mm) located on the right side of the bladder. Color Doppler flow imaging (CDFI) displayed rich colorful blood flow signals as a colored mosaic pattern in the same lesion, and spectral Doppler showed continuous turbulent flow in the systolic and diastolic phases with a high-velocity low-resistance waveform (Figure 1). In the context of these imaging features, pelvic AVM was suspected.

**Figure 2:**
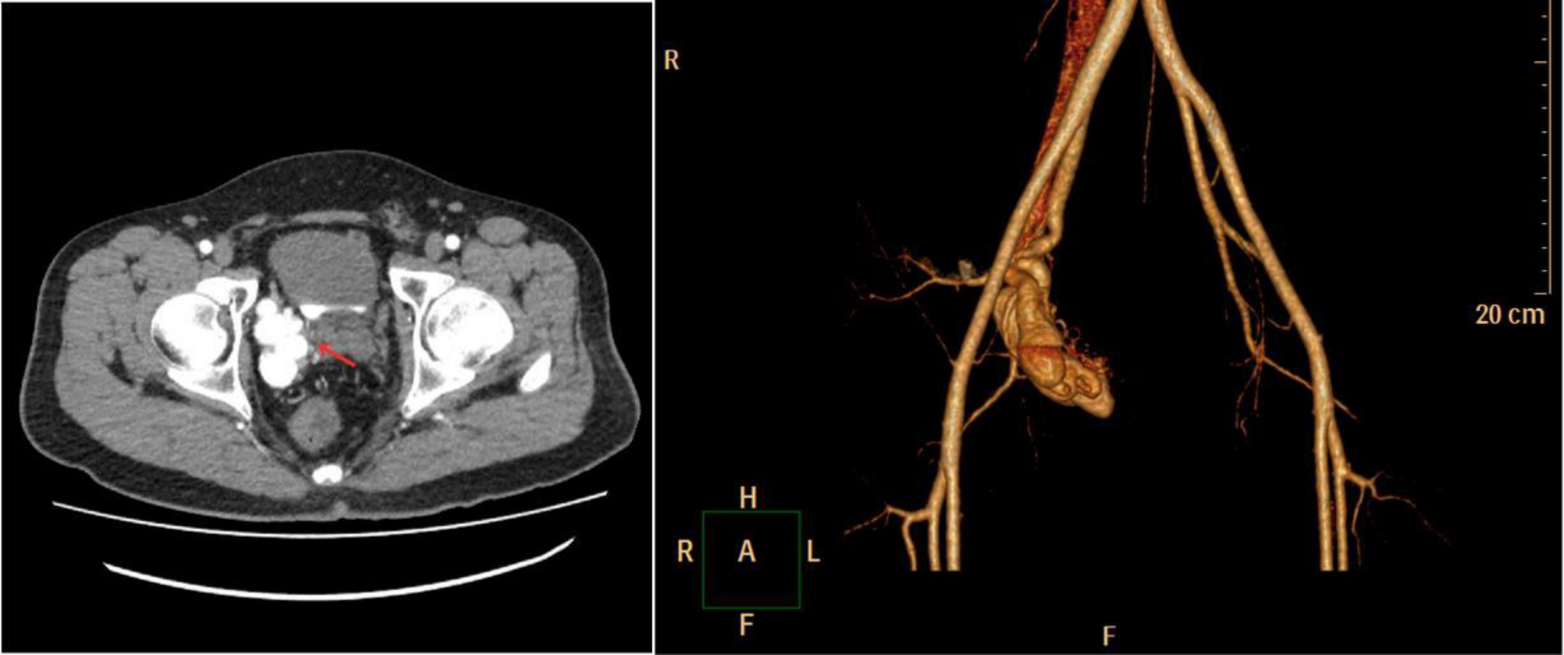
Computed tomographic angiography showed a large pelvic arteriovenous malformation (red arrow) supplied mainly by the right internal iliac artery.

**Figure 3:**
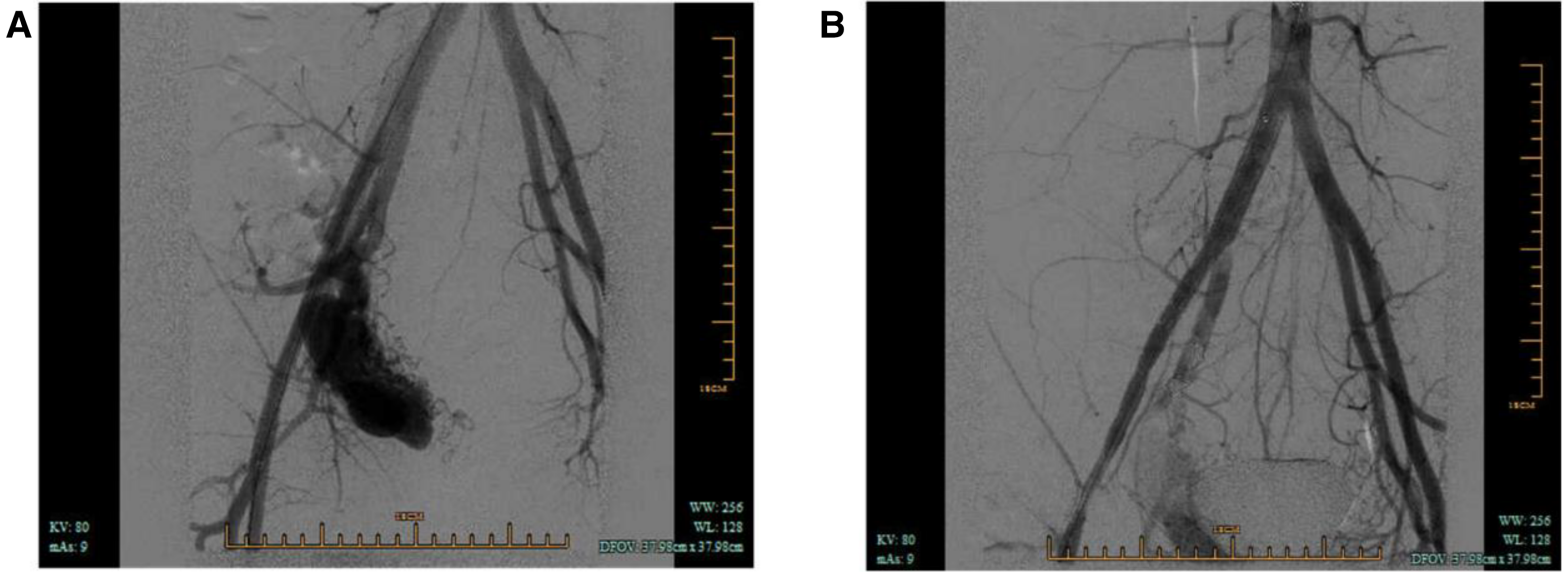
(**A**) Arteriography demonstrated pelvic arteriovenous malformation and feeding arteries arising from the right internal iliac artery. (**B**) Arteriography postembolization.

**Figure 4:**
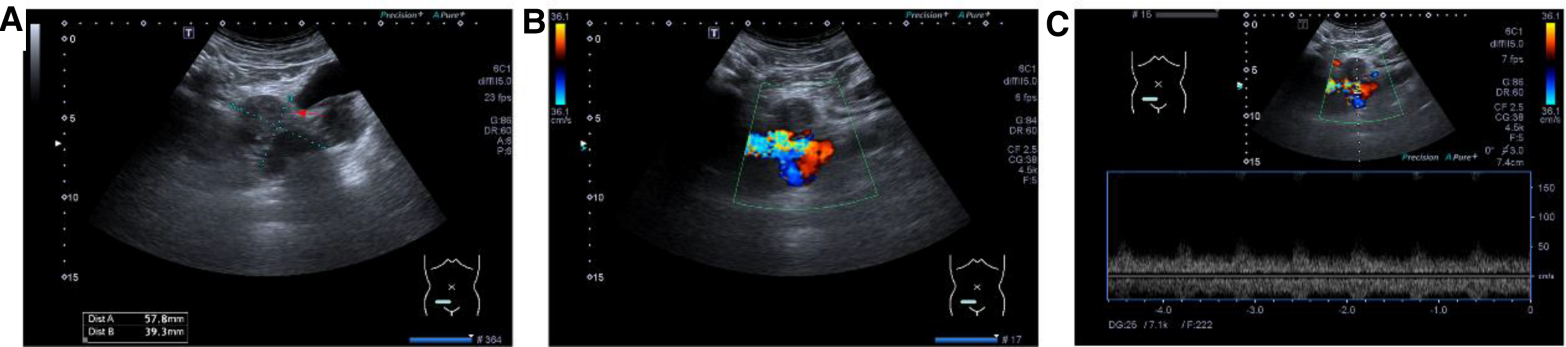
(**A**) Part of the irregularly shaped anechoic lesions became hypoechoic (red arrow) in the B-mode ultrasound. (**B**) Color Doppler flow imaging showed the colored mosaic pattern decreased in volume. (**C**) Spectral Doppler showed the systolic blood flow velocity is reduced after embolization.

The patient underwent computed tomographic angiography (CTA) after the sonographic diagnosis of pelvic AVM, whereby a large pelvic AVM supplied mainly by the right internal iliac artery was found (Figure 2), thus confirming the final diagnosis of pelvic AVM. He was then admitted for transcatheter embolization. The preoperative laboratory examinations were within normal limits. When arteriography indicated feeding arteries arising from the right internal iliac artery, the right internal iliac artery was embolized by spring coils (Figure 3). The patient recovered uneventfully after embolization. He remained asymptomatic during the follow-up period and was re-examined with abdominal US 19 months after embolization, in which the pelvic AVM decreasing in volume and the systolic blood flow velocity reduction were revealed (Figure 4).

## Discussion

Pelvic congenital AVMs in males are relatively rare. In 2002, Game et al. (2) reported 2 cases of pelvic congenital AVMs in male patients and reviewed 15 previously published cases, after which only 8 more cases (listed in Table 1) have been reported to date. Here, we present a rare case report of a male congenital pelvic AVM diagnosed by abdominal US with a detailed description of the US findings, hoping this work may contribute to the ultrasonic diagnosis of congenital pelvic AVMs.

The malformed vascular plexus formed by the remnant capillary network constitute the structures between the arterial and venous systems. AVM may occur in any part of the body but mainly in the brain, neck, lung, and kidney. Pelvic AVMs are relatively rare, especially in males. The presenting symptoms of pelvic AVM are nonspecific and can be either subtle or dramatic and may even be fatal (13). Both the low incidence and low specificity of the symptoms make the early clinical diagnosis difficult. Although palpating a pulsatile mass and hearing loud or harsh noises on physical examination is a significant indication of pelvic AVM (2), not all masses may be palpable, and rectal examination is not routine. In addition, AVM might not be diagnosed during routine examination (14), contributing to a diagnostic dilemma.

Despite the fact that abdominal US is usually the initial routine imaging examination, according to previous literature reports, the diagnostic value of US remains questionable with regard to male congenital pelvic AVM. Game et al. (2) considered that US could only show a nonspecific hypoechogenic area and was not able to differentiate an AVM from a cyst or an abscess. Aslan et al. (14) suggested that the US characteristics of AVM are nonspecific but could alert the sonographer to the possibility of a vascular abnormality. Although previous literature has reported congenital AVM diagnosed by transrectal US in which a pulsating mass was palpable on rectal examination, the US features of AVM were not described in detail (5). Moreover, in most previous articles, US only detected the lesions while being unable to make the diagnosis of AVM (6, 12).

In contrast to previous literature, we believe that, with the application of Doppler, US is also a potential tool of solid diagnostic value for congenital pelvic AVM in males. Mittal et al. (15) considered that US and Doppler imaging could be used to diagnose vascular anomalies. In the present case, we found multiple tortuous tubular structures within an irregularly shaped anechoic lesion on gray-scale ultrasonography. To help differentiate this from a cyst or abscess, CDFI was also performed, whereby rich colorful blood flow signals were revealed in the same lesion; by contrast, cysts or abscess cavities lack the presence of vascularity and color flow. Aneurysms and pseudoaneurysms share some characteristics with AVM on US because they are all hypervascularized, but they also have their own unique features (as listed in Table 2) and can be differentiated by US.

**Table 2:** Key points of differential diagnosis by US in pelvic cystic masses.

	Two dimensional	Color Doppler	Spectral Doppler
Cyst/abscess	Regular-shaped anechoic lesion	No blood flow signals	No blood flow signals
Aneurysm	Saccular or fusiform dilatation of an artery and the structure of arterial wall is complete	Red-blue blood flow signals	Low-velocity, swirling blood flow
Pseudoaneurysm	Pulsatile hematomas that communicate with an artery through a localized arterial disruption, sometimes shows hypoechoic areas because of thrombosis	Color flow jet area in the localized arterial disruption	Bi-directional turbulence in the systolic and diastolic phases in the localized arterial disruption
Arteriovenous malformation	Multiple tortuous tubular structures within an irregular-shaped anechoic lesion	Rich colorful blood flow signals as a colorful mosaic pattern	Continuous turbulent flow in the systolic and diastolic phases with a high-velocity low-resistance waveform

US, ultrasound.

The treatment options of congenital pelvic AVM in males depend on the severity of presenting symptoms, and asymptomatic or mildly symptomatic lesions are considered not to require therapy (2). A variety of therapies, including ligation of the afferent arteries, excision of the lesion, and embolization, have been used to treat AVM in male patients. However, a surgical approach has been reported to be usually unsuccessful and complicated by hemorrhage, adjacent organ damage, and recurrence (16, 17). In contrast, a lower rate of morbidity, mortality, and invasiveness makes embolization the preferred treatment (18). The goal of embolization is the elimination of the AVM nidus (12). Various embolic materials have been used to treat AVM lesions, including metallic coils, absolute ethanol, ethylene vinyl alcohol copolymers, and rapidly polymerizing acrylic adhesives (18–20). Improved catheters, the use of superselective techniques, and liquid embolic agents have been reported to be effective in treating AVMs, which may provide the best long-term result (18, 21, 22). The patient in our study complained of progressive pain in the right lower abdomen and lumbar area, which eventually became unbearable and could not be relieved by rest. Because he has not yet completed his childbearing and strongly desired fertility despite the risk of recurrence, we used coil embolization of the right internal iliac artery, which only relieved the symptoms but not eliminated the AVM nidus. Therefore, a tight follow-up is essential, and the patient may need further treatment after his childbearing.

## Conclusions

We presented the case of a male congenital pelvic AVM first diagnosed by abdominal US. Furthermore, we described the ultrasonic features of AVM in detail and established some key points of differential diagnosis by US in pelvic cystic masses, rarely mentioned in previous literature. For pelvic cystic masses found on two-dimensional US, color Doppler and spectral Doppler are necessary for further differential diagnosis. Furthermore, congenital pelvic AVM should be included in the differential diagnosis of pelvic cystic masses in males despite its low incidence.

## Data Availability

The original contributions presented in the study are included in the article/Supplementary Material, further inquiries can be directed to the corresponding authors.
